# Enhancing Surgical Safety: Evaluating Compliance With the WHO Checklist in a Peripheral Hospital in Sri Lanka

**DOI:** 10.7759/cureus.79615

**Published:** 2025-02-25

**Authors:** NJASS Jayasuriya, BM Munasinghe, Nishanthan Subramaniam, Sameera Prasanga Lokuliyana, DMP Withanage, KAL Ravihari

**Affiliations:** 1 Anaesthesiology and Critical Care, Base Hospital, Thambuththegama, LKA; 2 Anaesthesiology, Mackay Base Hospital, Queensland, AUS; 3 Anaesthesia, Wrexham Maelor Hospital, Wrexham, GBR

**Keywords:** compliance, patient safety, resource-limited settings, surgical safety checklist, who

## Abstract

Background

The World Health Organization (WHO) Surgical Safety Checklist is utilised globally to enhance communication and teamwork, ensuring patient safety in surgeries and reducing complications and mortality. Despite its efficacy, compliance with the checklist may be variable, particularly in resource-limited settings. This audit evaluated the adherence to the WHO Surgical Safety Checklist in the surgical theatre of Base Hospital Thambuththegama, Sri Lanka, to identify the gaps and areas for improvement.

Methods

A prospective audit assessed 102 surgical cases over three weeks, including elective and non-elective procedures. Data collection involved real-time observation of checklist use and assessment of staff participation. Compliance was evaluated based on checklist attachment, completion, and timing, while staff participation rates were analysed across roles.

Results

In 87.25% of cases, the checklist was attached to the bed head ticket (BHT). Fully completed checklists were observed in only 34.31% of cases, with just 13.73% completed at the correct time. Staff participation was highest among anaesthetists (90.5%) and house officers (88.1%) but significantly lower among consultants (7.1%). Key barriers included incomplete sections, incorrect timing, and inconsistent participation from senior staff.

Conclusions

The audit highlights critical gaps in compliance with the WHO Surgical Safety Checklist, emphasising the need for targeted interventions. These include staff training, real-time monitoring, and enhanced accountability. Addressing these gaps can significantly improve surgical safety and patient outcomes. A re-audit is planned to evaluate the impact of the proposed changes.

## Introduction

The surgical safety checklist has been widely recognised as a critical tool for enhancing communication, patient safety, and surgical outcomes. Since introducing the World Health Organization (WHO) Surgical Safety Checklist in 2008, numerous studies have demonstrated its efficacy in reducing surgical complications, including infections, mortality, and postoperative care errors [1-3]. These findings underscore the importance of standardised checklists in fostering effective communication and team coordination during surgical procedures.

Despite these proven benefits, challenges persist in adopting and adhering to checklist protocols, particularly in resource-limited settings. Factors such as time constraints, lack of awareness, inadequate training, and workflow interruptions often contribute to inconsistent use and improper timing of checklist completion [4]. It is important to note that the effectiveness of checklists is significantly influenced by proper timing and team compliance [5].

Preliminary observations at Base Hospital in Thambuththegama, Sri Lanka, indicated variability in checklist usage, with some cases having incomplete or missing checklists and others failing to adhere to the correct timing. Such lapses can compromise patient safety, increase the risk of preventable errors, and affect team accountability. This study aimed to assess our institution's current adherence to surgical safety checklist protocols and to identify gaps in compliance and areas for improvement.

The objectives of the study were to measure the current rate of compliance with the WHO Surgical Safety Checklist, identify barriers to full compliance with the checklist, compare current practice with established standards, and provide recommendations to enhance the implementation of the checklist in the surgical theatre.

## Materials and methods

This was a prospective clinical audit at Base Hospital in Thambuththegama, Sri Lanka. Related data of all surgical procedures (elective and non-elective) conducted in the main surgical theatre over three weeks from November 1, 2024, were collected. The operation theatre (OT) staff were informed about the audit in advance; however, the exact period was not disclosed. Even if this were anticipated and they changed their practices, it would illustrate that they understood the importance of conducting the checklist, increased compliance, and improved patient safety, the ultimate goal of the study.

The inclusion criteria were elective and non-elective surgical procedures conducted during the audit period in the main surgical theatre, including obstetric, gynaecological, and general surgical cases. Procedures performed under general or spinal anaesthesia were included. Minor surgical interventions or outpatient procedures conducted outside the main surgical theatre and immediate surgical interventions due to life-threatening conditions making checklist completion impractical due to critical time constraints were excluded. Standard WHO Surgical Safety Checklists are completed on paper in our hospital. Study data were collected in the surgical theatre by observing procedures and reviewing bed head tickets (BHTs) to assess the presence and completeness of the WHO Surgical Safety Checklist. Each section (sign-in, time-out, sign-out) was evaluated for adherence to WHO guidelines, timing, and completeness while documenting the staff members involved in its completion. The data were organised in Microsoft Excel (2021; Microsoft Corp., Armonk, NY), with percentages and frequencies calculated to assess compliance rates, timing accuracy, and staff participation. Key findings were visualised using graphical representations.

All data were anonymised, excluding identifiable information. The written approval of the institution's head was obtained, and the relevant consultants of surgical departments, the OT sister-in-charge, and the surgical team members were informed before the study via a written datasheet. As the patients or surgical team members were not contacted, and only the secondary data were collected using the checklist data, no ethical clearance was required for this audit. Only existing documentation was reviewed without interfering with patient care or surgical workflows, ensuring the audit posed no risk to patients. The previous year's caseload in the OT, where the audit was conducted, was around 1500, for which the checklist was required to be completed. Out of that, the current audit assessed 6.8% of the cases (n=102), thus reliably representing the overall sample.

## Results

Data sample overview

**Figure 1:**
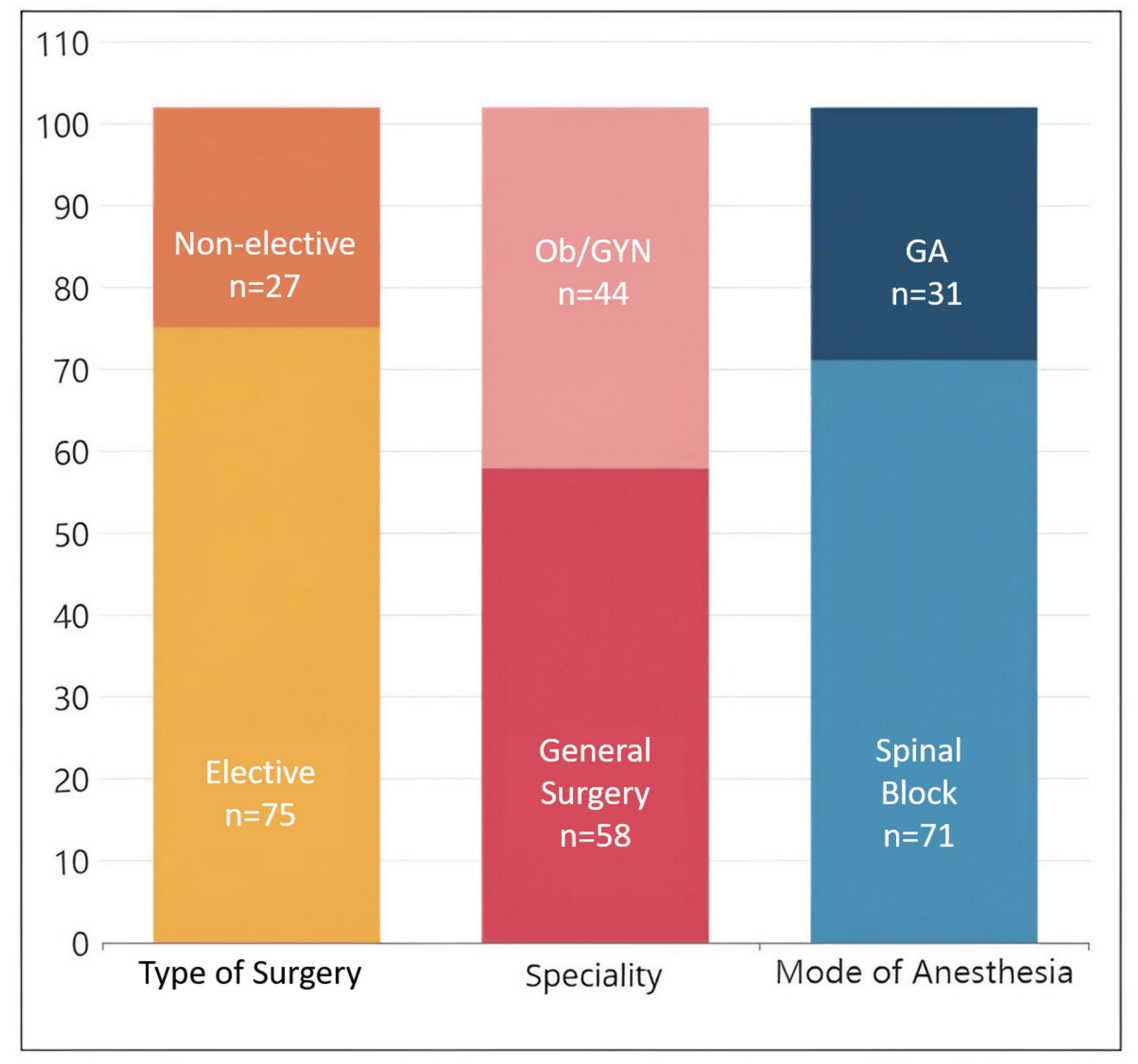
Data sample overview OB/GYN: obstetrics and gynaecology; GA: general anaesthesia

The total number of analysed cases was 102. Among these, the age distribution was as follows: <20 years, seven (6.86%); 20-39 years, 41 (40.20%); 40-59 years, 39 (38.24%); and >60 years, 15 (14.71%). Regarding sex distribution, 63.73% (n=65) of the cases were female patients, and 36.27% (n=37) were male patients. Elective surgeries accounted for 73.53% (n=75) of cases, and non-elective surgeries for 26.47% (n=27). General surgical cases comprised 56.86% (n=58), while obstetric and gynaecological cases constituted 43.14% (n=44). Spinal anaesthesia was performed in 69.61% (n=71) of cases, and general anaesthesia was administered in 30.39% (n=31) (Figure 1). During the study period, other modes of anaesthesia were not administered.

**Figure 2:**
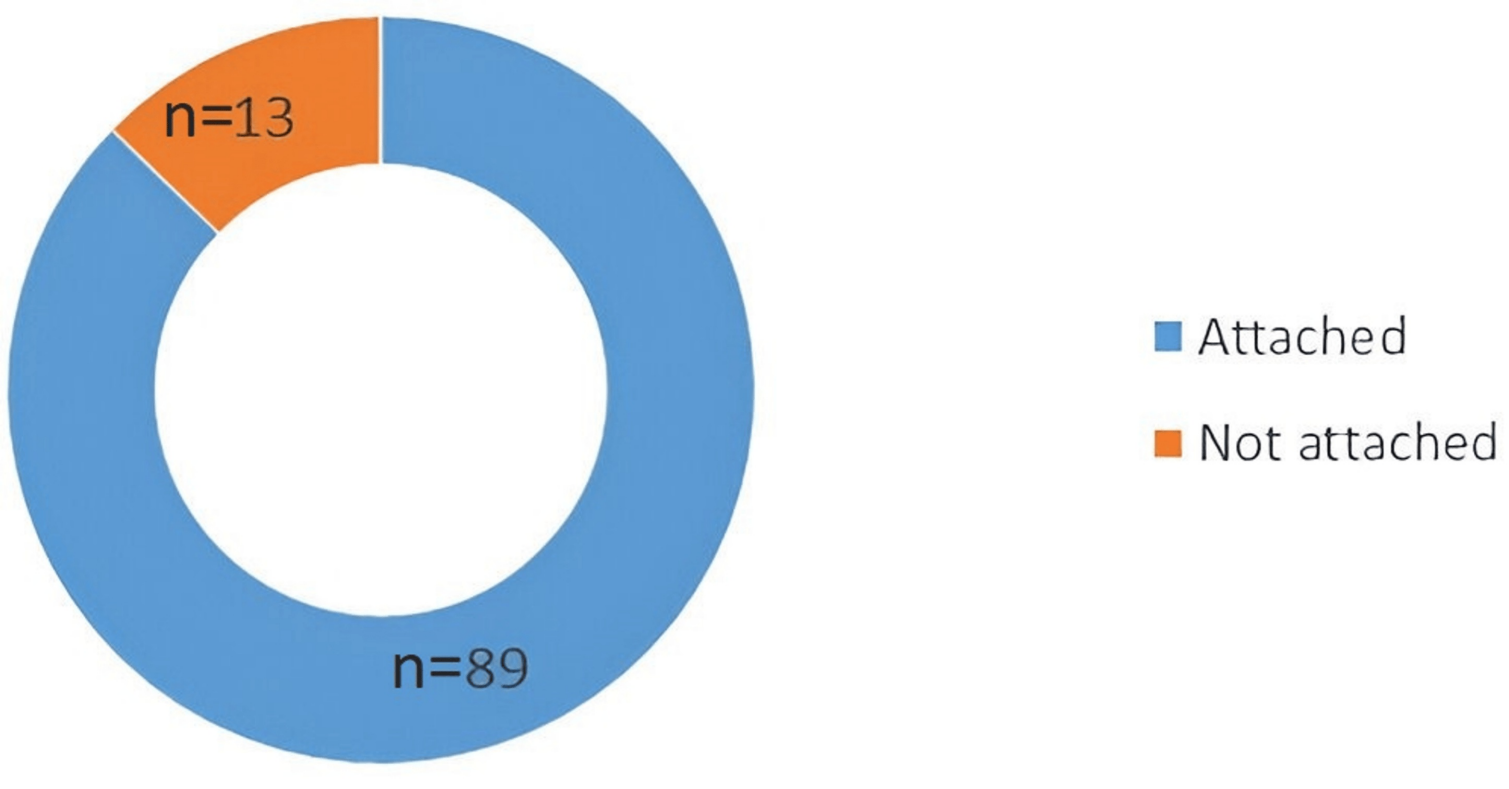
Details of checklist attachment to bed head tickets (BHTs)

The details of the practice of attaching the checklist to the patient's BHT are illustrated in Figure 2.

**Figure 3:**
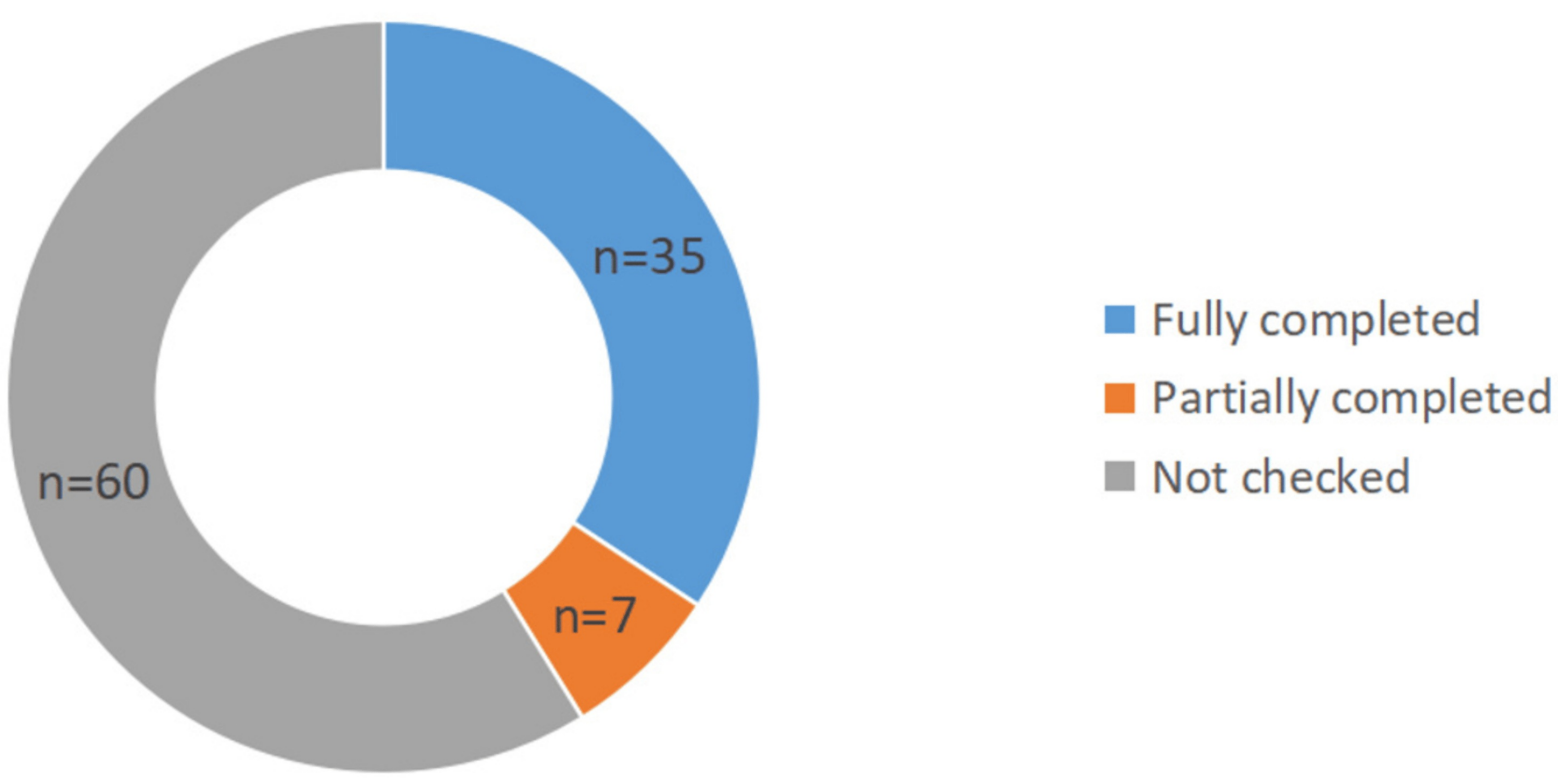
Checklist completion statistics

The number of checklists attached to the BHTs was 89 (87.25%), and no checklists were attached to 13 (12.75%) BHTs. The details of checklist completion practices are depicted in Figure 3.

**Figure 4:**
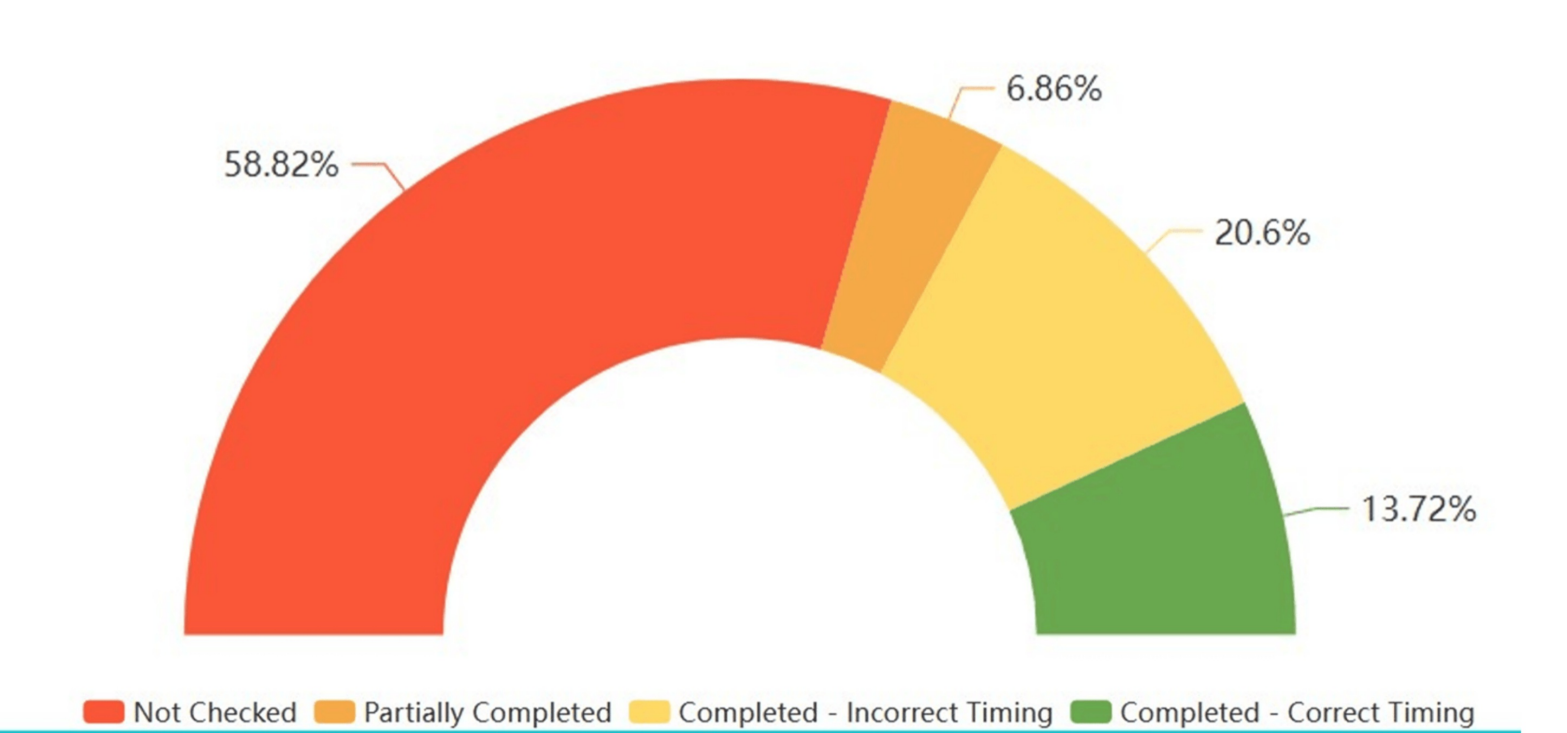
Overall checklist completion statistics

The number of fully completed checklists was 35 (34.31%). The number of partially completed checklists was seven (6.86%). The number of checklists that were not completed was 60 (58.82%). Among the fully completed checklists (n = 35), the correct timing of each stage, namely, sign-in, time-out, and sign-out, was accurately monitored (Figure 4).

**Figure 5:**
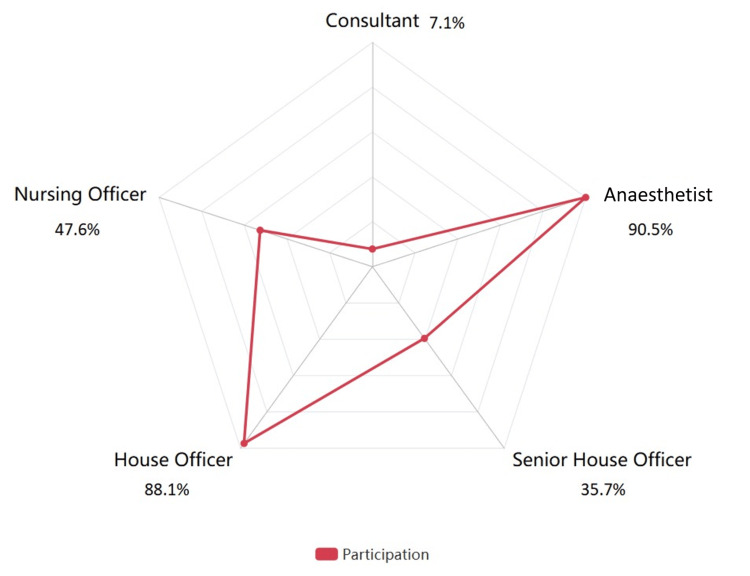
Analysis of staff participation

Checklists that were fully completed but those completed at an incorrect timing were 21 (20.58%). These include checklists where all three sections were completed, but all were completed at sign-in instead of their correct times. Fully completed checklists at the correct timing were 14 (13.73%). These are the checklists where all three sections, sign-in, time-out, and sign-out, were completed at the correct time. Analysis of staff participation in checklist completion is depicted in Figure 5.

The data show total staff participation in fully and partially completed checklists across different roles (n=42). 

## Discussion

The analysis revealed that out of 102 BHTs, only 87.25% (n=89) had checklists attached, while 12.75% (n=13) lacked this critical document. While most BHTs included checklists, the fact that 100% compliance was not achieved is a significant concern, as it compromises patient safety and the quality of surgical care [6]. Critical steps may be missed, potentially leading to adverse events. Missing documentation can also raise medico-legal issues, particularly when complications or adverse outcomes arise.

High patient volumes and staff shortages may lead to checklist omissions in low-resource institutions, particularly in non-elective cases. Staff may underestimate the importance of attaching the checklists due to a lack of awareness. Inconsistent monitoring or unclear accountability for checklist management inwards may also contribute to this. The checklist completion and timing analysis reveal significant gaps in adherence to proper protocol. A complete checklist is essential to address all critical safety checks across the surgical workflow. However, only about one-third of the checklists (34.31%) were fully completed, while more than half (58.82%) had no completed checklists, highlighting a significant compliance issue. Partially completed checklists (6.86%) indicate a lack of diligence in documentation. It may reflect incomplete communication among team members or interruptions during the workflow. These partially filled checklists are particularly concerning as they may leave critical safety checks unverified.

The absence of checklists is a significant safety concern. This indicates that an essential safety tool is bypassed in more than half of the cases. This could lead to many issues, such as an increased likelihood of preventable errors, lack of accountability in case of adverse outcomes, and errors in consistent, team-wide communication. Among the 35 fully completed checklists, 27.45% (n=21) were completed entirely at the sign-in stage, neglecting the critical time-out and sign-out stages. This is a wrong practice, as each section (sign-in, time-out, and sign-out) must be completed at a specific time. This reflects a poor understanding of the checklist process and undermines its intended safety purpose. Only 13.73% (n=14) of cases followed the correct WHO protocol by completing all three sections at the appropriate times. This low compliance for correctly completing all three phases demonstrates a significant failure to integrate the checklist process into the surgical workflow. The high percentage of incomplete or incorrectly timed checklists may point to issues with time management and workflow integration. Surgical teams may face pressure to expedite cases, leading to checklist shortcuts [7]. Without adequate training, checklists may be perceived as a bureaucratic requirement rather than a safety tool. The results may also reflect deeper cultural or systemic issues, such as resistance to change or the absence of a safety-first mindset [5].

The analysis of role participation in completing surgical safety checklists reveals critical insights into team members' engagement levels in adhering to safety protocols. The disproportionate participation rates suggest an uneven distribution of responsibility, which may undermine collective accountability. Effective teamwork requires all roles to actively contribute to checklist adherence to ensure comprehensive safety measures [8]. Consultants participated in only 7.1% (n=3) of cases, reflecting their minimal direct involvement in checklist management. This may be due to their focus on oversight rather than operational tasks. Their limited involvement in checklist completion may reflect a delegation of routine tasks to junior staff and signal a need for greater emphasis on consultant-level engagement in safety practices [9]. Anaesthetists participated in 90.5% (n=38) of cases, the highest among all roles. These data emphasise their central role in ensuring procedural compliance and patient safety. Their consistent involvement reflects a deep integration of checklist protocols into their workflow. House officers participated in 88.1% (n=37) of cases, closely following anaesthetists in active participation, reflecting their significant role in checklist completion. Senior house officers participated in 35.7% (n=15) of cases, showing lower participation rates than other medical staff. This could also result in missed opportunities for senior oversight during critical phases of surgery. This may be due to their reduced engagement with routine documentation and overlap in responsibilities with house officers during key stages of surgery. Even though nursing officers are often responsible for crucial checklist tasks, they only participated in 47.6% (n=20) of cases, which suggests a need for increased engagement, considering their central role in the surgical workflow [10]. This would cause safety concerns, leading to gaps in essential tasks, such as instrument counts and patient handover checks. The moderate rate may indicate variability in role assignments or responsibilities among nurses due to time constraints and staff shortages.

Following its global adoption in 2008, Sri Lanka officially implemented the WHO Surgical Checklist on December 24, 2013, to ensure effective integration into surgical workflows [11]. However, checklist adherence remains sub-optimal in studied settings [12]. Compared with global data, this audit revealed significant gaps in checklist completion. The compliance rate of 13.73% (n = 14) is considerably lower than the studies conducted in Pakistan (61.8%) and France (91%), indicating significant concerns about proper checklist usage [13]. The 34.3% completion of the checklist is lower than France (61%), and the reported rate is above 50% in middle and low Human Development Index (HDI) countries [13, 14]. However, the unavailability of checklists (12.75%) was much less in our institution, while in some resource-limited settings, this amounted to 25% [14]. This suggests that non-compliance is mainly due to training deficits and poor enforcement rather than resource limitations. In comparison to completion of only 13.73% at the correct timings in the sign-in, time-out, and sign-out phases, studies in Switzerland (96% to 100% for time-out) and Australia (82.4% in sign-in) show better adherence to each phase [15-16]. A notable global trend is the neglect of the sign-out phase, which is not only seen in our audit (13.73%) but also in studies from Australia (0%) and Switzerland (22%) [15-16]. This highlights that the preoperative and intraoperative checks are prioritised while postoperative checks are overlooked.

Studies from South Asia demonstrate that checklist completion has significantly improved after proper introduction and training [17]. It was also noted that there was a clear improvement in surgical outcomes after checklist implementation [17]. A study conducted in Sri Lanka found that although staff members were aware of the checklist, only a few had received formal training in checklist use [12]. These comparisons underscore that implementing lessons from high-performing institutions, such as mandatory compliance policies, training programs, consultant-led safety initiatives, and continuous monitoring, is essential to align our surgical safety practices with international standards and improve patient outcomes [18].

Limitations

The study period was short, and the assessed caseload was relatively small. This was mainly due to the investigators' clinical commitments and time constraints. Lengthening the study period would have obviated this, making the findings more generalisable. As the study participants (surgical team members) were informed that their compliance was being assessed, they may have modified their behaviour, leading to the Hawthorne effect (altering behaviour due to awareness of being observed). Knowledge of the study may have artificially increased compliance during data collection. Although this observation could have contributed to a marginal increment in checklist completion, the final results contradict this. This highlights that the surgical team members were unaware of the conduct of the study during that specific time frame despite their knowledge of its 'possible' conduct at some point.

Recommendations and action plan

Improving Checklist Adherence

Mandate 100% checklist completion with proper timing, integrating it into the preoperative verification process. Ensure blank checklists are available in the theatre and enforce strict policies requiring completed checklists for all cases. Emphasise correct timing for sign-in, time-out, and sign-out, embedding it into the surgical workflow with visual aids as reminders.

Enhance Role Participation

Encourage the involvement of consultants, senior house officers (SHOs), and nurses in checklist completion, emphasising the consultants' leadership role. Make checklist adherence a key performance indicator for senior staff and assign clear responsibilities to ensure accountability at every stage.

Implement Training Programs

Conduct regular training sessions for all staff to emphasise the importance of checklists, using case-based learning and simulations to demonstrate their impact on patient safety. Provide targeted training for nurses and SHOs to clarify their responsibilities and the critical role of checklists.

Introduce Monitoring and Feedback

Conduct regular audits to monitor compliance and identify areas for improvement. Use audit results to provide targeted feedback, reinforce positive behaviour, and address low compliance rates. Assign a dedicated team member, such as a circulating nurse, to ensure checklist completion and attachment.

Reaudit

Conduct a reaudit six months after implementing the action plan to assess improvements in checklist completion, timing adherence, and role participation. Use findings to address gaps and refine the action plan.

## Conclusions

The absence of checklists attached to BHTs is a critical gap that requires immediate attention. Even in the presence of checklists, there is significant non-compliance with checklist protocols. Completing the checklists in inappropriate stages poses patient safety issues. Participation in checklist adherence varied widely according to the role in our study. The absence or incorrect use of checklists compromises patient safety, teamwork, and surgical outcomes, highlighting the need for urgent corrective action. Continued training on the correct use, joint teamwork of multiple stakeholders, and regular auditing and constructive feedback at local and national levels would aid the sustained utility of this evidence-based practice.
